# Epidemiological characteristics and management of Gram-negative bacteraemia in different immunocompromised hosts: Observational single-center study

**DOI:** 10.1371/journal.pone.0327535

**Published:** 2025-07-07

**Authors:** Alice Toschi, Renato Pascale, Dino Gibertoni, Riccardo Pasquali, Andrea Grechi, Irene Grassi, Marta Malosso, Ludovica Mangione, Cecilia Bonazzetti, Beatrice Tazza, Matteo Rinaldi, Armando Amicucci, Caterina Campoli, Zeno Pasquini, Simone Ambretti, Pier Giorgio Cojutti, Francesca Bonifazi, Pierluigi Viale, Maddalena Giannella

**Affiliations:** 1 Department of Medical and Surgical Sciences, Alma Mater Studiorum University of Bologna, Bologna, Bologna, Italy; 2 Infectious Diseases Unit, Department for Integrated Infectious Risk Management, IRCCS Azienda Ospedaliero-Universitaria di Bologna, Bologna, Italy; 3 Epidemiology and Statistics Unit, IRCCS Azienda Ospedaliero-Universitaria di Bologna, Bologna, Italy; 4 Microbiology Unit, IRCCS Azienda Ospedaliero-Universitaria di Bologna, Bologna, Italy; 5 SSD Clinical Pharmacology, IRCCS Azienda Ospedaliero-Universitaria di Bologna, Bologna, Italy; 6 Haematology Unit, IRCCS Azienda Ospedaliero-Universitaria di Bologna, Bologna, Italy; Pescara General Hospital, ITALY

## Abstract

**Importance:**

Patients with Gram-negative bloodstream infections (GN-BSI) are classified as non-immunocompromised (n-IC) or immunocompromised (IC). However, immunosuppressive condition should not be considered univocally.

**Objective:**

To investigate epidemiological characteristics, management and outcome of GN-BSI in IC and non-IC patients.

**Methods:**

Retrospective single-center study of hospitalized patients with GN-BSI conducted over a 7-year period. Patients with GN-BSI were divided in: solid organ transplant (SOT) recipients, patients with hematologic malignancy (HM), patients with metastatic solid cancer (mSC), and non-major IC patients (nm-IC).

**Results:**

3544 patients analysed: 76.7% nm-IC, 6.5% SOT, 8.0% HM and 8.8% mSC. SOT and HM patients were younger (SOT: 56.6 ± 13.1 years; HM: 56.4 ± 14.5; nm-IC: 72.4 ± 16.1; mSC: 68.6 ± 13.1, p < 0.001) and had lower CCI value (SOT: 4.5 ± 2.4; HM: 4.1 ± 2.1; nm-IC: 5.5 ± 2.6; mSC: 9.7 ± 2.5, p < 0.001). Urinary tract infection was the most common source of BSI in nm-IC (nm-IC: 50.1%, HM:15%; SOT: 33.3%; mSC: 25.9%, p < 0.001), intra-abdominal infection was the more frequent source among SOT and mSC (SOT:42.3%; mSC: 49.3%, nm-IC: 27.8%, HM:29%; p < 0.001). Primary BSI was the first cause of GN-BSI in HM (HM: 62.1%; SOT: 18.5%; nm-IC: 17.2%; mSC: 10.6%, p < 0.001). The lowest rate of death was observed in SOT and the highest in mSC (SOT 8.2%; nm-IC 13.4%; HM 14.9%; mSC 19.9%, p < 0.001). Relapse rate was highest in SOT (SOT: 18.8%; HM: 11.8%; NMIC: 7.2%; aST: 7.1%, p < 0.001). Follow-up bloodcultures were associated with a lower mortality only among NMIC (HR = 0.317, 95% CI 0.178–0.563, p < 0.001) and aST (HR = 0.198, 95% CI 0.058–0.673, p = 0.010). The role of treatment duration on relapse was not evident in any group, conversely receiving at least 7 days of treatment was associated with a lower risk of 90-day mortality in SOT and HM patients.

**Conclusions:**

The characteristics and outcome of GN-BSI are peculiar between specific IC categories, therefore a personalized management should be implemented.

## Introduction

In the last 15 years Gram negative bloodstream infection (GN-BSI) has emerged as a main infectious complication, especially among hospitalized patients and those with chronic underlying conditions, in which it is associated with high rates of morbidity and mortality [1]. The standardization of diagnostic and therapeutic management of GN-BSI is challenging [2–3] due to the heterogeneity of GN-BSI in terms of host characteristics, causative agents and susceptibility patterns. As regards host characteristics, patients are mainly classified as non-immunocompromised (n-IC) or immunocompromised (IC), including in this latter group a wide spectrum of conditions with different degree and duration of immunosuppression. However, immunosuppressive condition should not be considered univocally, due to the different pathogenetic mechanisms underlying the alteration of the immune system. Immunosuppression due to anti-rejection therapy in patients undergoing solid organ transplant is quite different from that caused by an altered function of the immune system typical of haematological patient or determined by chemotherapy in a patient with a solid neoplasm. Consequently, IC patients with GN-BSI may have different aetiologies, therapeutic needs and outcomes depending on the underlying condition.

Indeed, among solid organ transplant recipients, GN-BSI account for 6% to 45% of all BSI, with reported mortality rates ranging from 3% to 52% [4]. Similarly, in haematological neutropenic patients, bacteraemia caused by GN rods occurs in 25% to 74% of cases, with *Escherichia coli* and *Pseudomonas aeruginosa* being the primary etiological agents. Mortality rates in these patients are highly variable, ranging from 6% to 40% [5]. Finally, in patients with solid tumors, GN aetiology account for over 60% of BSI, with a mortality rate of approximately 20%. *Escherichia coli* is the most frequently reported microorganism (47%) [6].

However, current literature typically focuses on a specific IC condition, sometimes comparing these patients to the general population [4–6]. We deem that a comparison of epidemiological characteristics and outcome of patients with GN-BSI according with the underlying IC status may provide useful information about the potential need of a different approach to the diagnostic and therapeutic management.

Therefore, the aim of this study is to investigate bacteremia characteristics, aetiology distribution, management, and outcome in different types of IC patients with GN-BSI. In addition, the impact of follow-up bloodcultures (FUBCs) on all-cause 30-day mortality and duration of antibiotic treatment on 90-day relapse were assessed overall and in each population.

## Methods

### Study design and setting

Single-center retrospective observational study of adult patients with GN-BSI hospitalized at IRCCS Azienda Ospedaliero-Universitaria di Bologna, a 1450-bed tertiary-care hospital in Bologna, Italy, over a 7-year period (January 1^st^ 2013 to December 31^st^ 2019). Follow-up for mortality/relapse was 90 days after the index BCs (time 0 for all outcomes).

During hospitalization, patient management was determined by attending physicians and was not dictated by local protocol. Data were anonymously collected from 5^th^ May 2023–12^th^ June 2023 using hospital records and inserted in a dedicated REDCap electronic case report form (eCRF) hosted by IRCCS Azienda Ospedaliero-Universitaria di Bologna [7]. The study was conducted according to the declaration of Helsinki and Good Clinical Practice guidelines and was approved by the local Ethics Committee (n° 894/2021/Oss/AOUBo).

### Population

All adult (≥ 18 years) patients hospitalized and diagnosed with GN-BSI during the study period were screened for inclusion using local microbiology registries. The inclusion criteria were as follows: hospitalized patients aged ≥ 18 years; diagnosis of GN-BSI, defined as one or more positive BCs obtained for ruling out an infection. The exclusion criteria were: patients receiving palliative care; unavailability of study data, particularly missing information regarding the type of pathogen, management, and outcomes. Patients with multiple episodes of infection were considered only once, at the time of first episode (index BCs).

Patients were classified into IC patients and non-major immunocompromised (nm-IC). IC patients were defined as patients with a reduced immune function and belonging to one of these three groups according to their underlying condition: solid organ transplant (SOT) recipients, patients with hematologic malignancies (HM) and patients with metastatic solid cancer (mSC) as recorded by Charlson comorbidity index [8] **(full definitions of the different CI conditions are provided in** S1 File). Conversely, non-major immunocompromised (nm-IC) patients were defined as patients without the previous conditions, including both patients with no underlying immunocompromising condition and patients with minor immunocompromising conditions (e.g., advanced age, chronic renal insufficiency, liver cirrhosis).

If patients had more than one major immunocompromising condition, the most clinically relevant one at the time of index BSI was considered, according to authors’ judgment.

### Variables and definitions

Primary endpoint was all-cause 30-day mortality. The secondary endpoint was infection relapse within 90 days, defined by a positive BC for the same pathogen of the index BC after treatment course was completed with clinical cure. Clinical cure was defined as the resolution of all signs and symptoms of infection according to vital signs, evolution of SOFA score and laboratory data, assessed at 7 days from index blood cultures BCs. FUBCs were defined as BCs drawn between 48 hours and 7 days after index BCs. Results of FUBCs were classified as positive for the same pathogen; positive for a different pathogen from that of index BCs; and negative. Duration of active antibiotic therapy was defined as the number of consecutive days during which the patient received an appropriate antibiotic regimen. A detailed description of variables and definitions is reported in S1 File.

### Statistical analysis

Patients’ characteristics were described as absolute and relative frequencies. Continuous variables were summarized with mean and standard deviation if normally distributed or with median and interquartile range (IQR) if non-normally distributed. Comparison of patients’ characteristics across the four subpopulations of IC and nm-IC patients was performed using ANOVA, Kruskal-Wallis test or chi square test, as appropriate. The post-hoc comparison for statistically significant tests was carried out by Sidàk post hoc test (after ANOVA), Holm post-hoc test (after Kruskal-Wallis test) applying alpha-level adjustment for family-wise error rate. After chi-square test, the categories having a Pearson residual greater > 2 (proportion greater than expected) and <−2 (proportion lower than expected) were identified as those contributing to the test’s statistical significance [9]. Survival analyses to identify predictors of 30-day mortality and 90-day relapse since the date of BSI were carried out using Royston-Parmar parametric survival models [10], because the proportionality of hazards assumption between patients with and without FUBC was not met. Several model parametrizations (exponential, log-logistic, probit) with 1–5 splines were checked, and among them the one providing the lower Bayesian Information Criterion (BIC) value was chosen. To remove immortal time bias, patients with FUBC entered the analysis at the date of FUBC (performed at a median time of 4 days after BSI). Moreover, patients who died within 5 days from BSI and without FUBC were removed from this analysis. When 30-day mortality was the outcome, FUBC performed within 7 days was set as the main exposure and as time-dependent variable. When 90-day relapse was the outcome, death was included as competing risk, survival time started at the end of antibiotic therapy course, and duration of therapy (< 7 days or ≥7 days) was the main exposure. The cumulative incidence of death and relapse was obtained according to the method described in Hinchliffe et al. [11], which entails including the two causes as both main effects and time-dependent effects, and creating an indicator for the interaction of each competing risk and levels of the exposure. In this way, a stratified model is obtained with separate baseline survival estimated for relapse and death. The multivariable models were performed first on the overall population and subsequently on each subpopulation separately, and included as potential confounders variables selected for their clinical interest. Confounders expressed as continuous variables (age, Charlson Comorbidity Index, SOFA score) were centred around their mean. Stata v.18.0 was used for all analyses; specifically, the stpm2 [12] and stpm2cif [11] packages were used for multivariable survival analysis.

## Results

**Fig 1:**
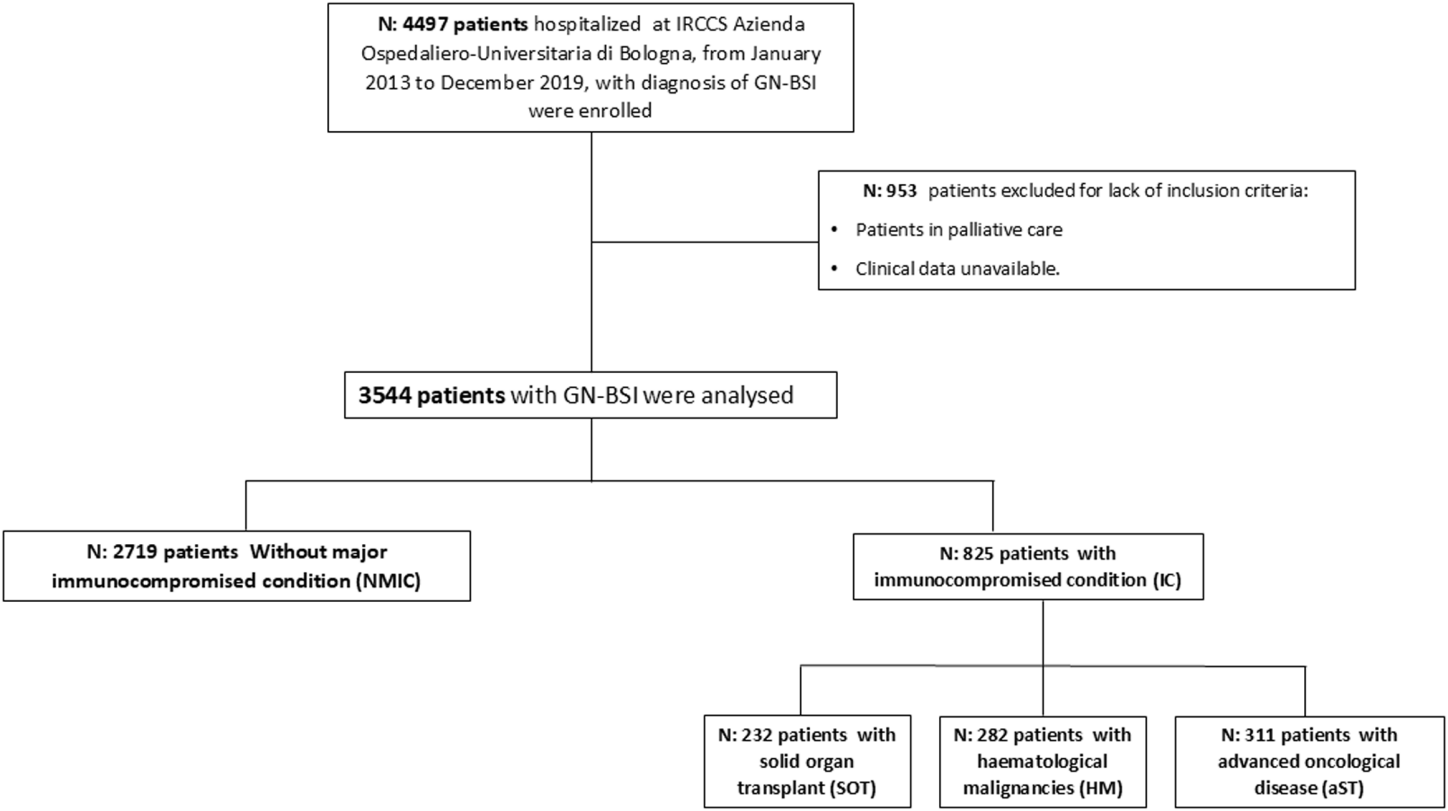
Study flow chart. **Legend**: Flow chart illustrating patient inclusion, exclusion criteria, and final stratification for analysis. GN-BSI = Gram negative BSI.

In the study period, 4497 patients were hospitalized with diagnosis of GN-BSI, among which 3544 were included in the study. Of these, 2719 (76.7%) were nm-IC, 232 (6.5%) SOT, 282 (8.0%) HM and 311 (8.8%) mSC (Fig 1).

As shown in Table 1, male gender was more frequent in the SOT group (SOT: 72.4% vs nm-IC: 53.7%; HM: 61.7%; mSC: 54.3%, p < 0.001). SOT and HM patients on average were younger (SOT: 56.6 ± 13.1 years vs HM: 56.4 ± 14.5; nm-IC: 72.4 ± 16.1; mSC: 68.6 ± 13.1, p < 0.001) and had lower CCI value than patients with mSC and nm-IC (SOT: 4.5 ± 2.4 vs HM: 4.1 ± 2.1; nm-IC: 5.5 ± 2.6; mSC: 9.7 ± 2.5, p < 0.001). CPE rectal colonization was observed more frequently in SOT and mSC patients (SOT: 33.6%; mSC: 20.6%; nm-IC: 13.0%; HM: 7.1%, p < 0.001). Septic shock at the time of GN-BSI onset was less frequently observed in HM (4.0%, p = 0.001). Specific characteristics of SOT, HM and mSC are summarized in S1–S3 Tables. Among secondary BSI, urinary tract infection (UTI) was the most common source of BSI in nm-IC, while intra-abdominal infection (IAI) was the more frequent source among SOT and mSC patients. Primary BSI was the first cause of GN-BSI in HM (HM: 62.1%; SOT: 18.5%; nm-IC: 17.2%; mSC: 10.6%, p < 0.001). Regarding aetiology, Enterobacterales were more common than non-fermentative Gram-negative (NFGN) rods. Among Enterobacterales, *Escherichia coli* was the leading pathogen (61.9%), followed by *Klebsiella* spp. (27.6%). NFGN rods were more frequently isolated in HM than other populations (18.8%, p < 0.001) and *Pseudomonas* spp. was the most common microorganism. SOT group had significant higher rate of resistance strains compared to all groups, except for FQR higher in HM group (72.7%, p < 0.001).

**Table 1:** Characteristics of the study population.

	N	A. nm-IC (n = 2719, 76.7%)	B. SOT (n = 232, 6.5%)	C. HM (n = 282, 8.0%)	D. mSC (n = 311, 8.8%)	Total Population (n = 3544)	Test, p*	Post-hoc
Age (years), median (IQR)	3543	76.0 (64.4-84.0)	58.1 (50.6-65.6)	59.1 (47.6-66.4)	70.2 (60.4-78.6)	72.7 (59.9-82.4)		
Age (years), mean±SD	3543	72.4 ± 16.1	56.6 ± 13.1	56.4 ± 14.5	68.6 ± 13.1	69.8 ± 16.5	152.6, < 0.001^	A > B C D; D > B C
Gender (male)	3544	1461 (53.7)	168 (72.4)	174 (61.7)	169 (54.3)	1972 (55.6)	34.8, < 0.001	B+
Underlying diseases								
Congestive heart failure	3544	527 (19.4)	22 (9.5)	3 (1.1)	24 (7.7)	576 (16.3)	91.8, < 0.001	A + , B-, C-, D-
Chronic kidney disease	3544	308 (11.3)	55 (23.7)	10 (3.5)	23 (7.4)	396 (11.2)	57.8, < 0.001	B + , C-, D-
Chronic obstructive pulmonary diseases	3544	504 (18.5)	11 (4.7)	20 (7.1)	30 (9.6)	565 (15.9)	61.1, < 0.001	A + , B-, C-, D-
Diabetes with organ damage	3544	173 (6.4)	19 (8.2)	2 (0.7)	11 (3.5)	205 (5.8)	20.3, < 0.001	C-
End stage liver disease	3544	262 (9.6)	89(38.4)	4 (1.4)	42 (13.5)	397 (11.2)	207.5, < 0.001	B + , A-, C-
Charlson Comorbidity index	3544	5.5 ± 2.6	4.5 ± 2.4	4.1 ± 2.1	9.7 ± 2.5	5.7 ± 2.9	310.5, < 0.001^	D > A B C; A > B C
Inpatient ward	3542						157.1, < 0.001	
Internal medicine		1852 (68.2)	151 (65.1)	271 (96.1)	230 (74.0)	2504 (70.7)		C+
Surgery		316 (11.6)	37 (15.9)	0 (0)	55 (17.7)	408 (11.5)		D + , C-
ICU		268 (9.9)	38 (16.4)	6 (2.1)	10 (3.2)	322 (9.1)		B + , C-, D-
Emergency Dept		281 (10.3)	6 (2.6)	5 (1.8)	16 (5.1)	308 (8.7)		A + , B-, C-, D-
Site of BSI acquisition	1948						94.6, < 0.001	
Community acquired		477 (32.1)	18 (13.9)	9 (5.4)	30 (18.1)	534 (27.4)		A + , B-, C-, D-
Healthcare associated		339 (22.8)	32 (24.8)	32 (19.0)	33 (19.9)	436 (22.4)		
Hospital acquired		669 (45.0)	79 (61.2)	127 (75.6)	103 (62.0)	978 (50.2)		C + , D + , A-
Rectal swab positivity for CPE prior bacteraemia	3537	352 (13.0)	78 (33.6)	20 (7.1)	64 (20.6)	514 (14.5)	179.2; < 0.001	B + , D + , A-, C-
Clinical severity at BSI onset								
SOFA score, median (IQR)	3544	3.55 ± 2.98	4.72 ± 3.29	4.46 ± 2.17	3.10 ± 2.62	3.66 ± 2.94	21.6, < 0.001^	B > A D, C > A D
Septic shock	3444	281 (10.6)	20 (8.9)	11 (4.0)	20 (6.6)	332 (9.6)	16.1, 0.001	C-
BSI sources	3543						338.4, < 0.001	
Primary		468 (17.2)	43 (18.5)	175 (62.1)	33 (10.6)	719 (20.3)		C + , A-, D-
Secondary		2250 (82.8)	189 (81.5)	107 (37.9)	278 (89.4)	2824 (79.7)	187.6, < 0.001	C-
Lung		218 (9.7)	17 (9.0)	23 (21.5)	15 (5.4)	273 (9.7)		C + , D-
Intra-abdominal		625 (27.8)	80 (42.3)	31 (29.0)	137 (49.3)	873 (30.9)		B + , D + , A-
UTI		1128 (50.1)	63 (33.3)	16 (15.0)	72 (25.9)	1279 (45.3)		A + , B-, C-, D-
Other		130 (5.8)	18 (9.5)	12 (11.2)	32 (11.5)	192 (6.8)		D+
CVC		149 (6.6)	11 (5.8)	25 (23.4)	22 (7.9)	207 (7.3)		C+
**Etiology**								
Polymicrobial BSI	3524	374 (13.8)	25 (10.8)	40 (14.3)	64 (20.6)	503 (14.2)	12.8, 0.005	D+
Enterobacterales	3544	2425 (89.2)	204 (87.9)	229 (81.2)	267 (85.9)	3125 (88.2)	17.4, 0.001	C-
Klebsiella spp		619 (25.5)	87 (42.6)	60 (26.2)	95 (35.6)	861 (27.6)		B + , D+
Escherichia coli		1530 (63.1)	98 (48.0)	160 (69.9)	145 (54.3)	1933 (61.9)		B-
Enterobacter spp		170 (7.0)	13 (6.4)	6 (2.6)	20 (7.5)	209 (6.7)		C-
Citrobacter spp		0 (0.0)	0 (0.0)	1 (0.4)	0 (0.0)	1 (0.0)		C+
Serratia spp		2 (0.1)	(0.0)	(0.0)	(0.0)	2 (0.1)		
Morganella spp		2 (0.1)	(0.0)	(0.0)	(0.0)	2 (0.1)		
Proteus spp		102 (4.2)	6 (2.9)	2 (0.9)	7 (2.6)	117 (3.7)		C-
Non-fermentative Gram negative	3544	293 (10.8)	28 (12.1)	53 (18.8)	44 (14.1)	418 (11.8)	17.7; 0.001	C+
Pseudomonas spp		191 (65.2)	23 (82.1)	46 (86.8)	34 (77.3)	294 (70.3)		
Acinetobacter spp		67 (22.9)	4 (14.3)	5 (9.4)	4 (9.1)	80 (19.2)		
Stenotrophomonas spp		35 (11.9)	1 (3.6)	2 (3.8)	6 (13.6)	44 (10.5)		
**Susceptibility pattern**								
ESCR	3544	952 (35.0)	119 (51.3)	112 (39.7)	122 (39.2)	1305 (36.8)	26.5; < 0.001	B+
FQR	3544	1129 (41.5)	128 (55.2)	205 (72.7)	129 (41.5)	1591 (44.9)	112.0; < 0.001	C + , B + , A-
BL/BLI R	3544	1196 (44.0)	143 (61.6)	148 (52.5)	151 (48.6)	1638 (46.2)	32.8; < 0.001	B+
Carbapenem-R	3544	319 (11.7)	61 (26.3)	33 (11.7)	45 (14.5)	458 (12.9)	41.3; < 0.001	B+
DTR	3544	219 (8.0)	53 (22.8)	20 (7.1)	36 (11.6)	328 (9.3)	59.2; < 0.001	B + , A-
**Management of BSI**								
Execution of FUBC	3544	561 (20.6)	69 (29.7)	83 (29.4)	83 (26.7)	796 (22.5)	17.8; < 0.001	B + , C+
FUBC result	1047						10.1; 0.122	
Positive for same pathogen		144 (19.5)	25 (28.4)	16 (14.8)	31 (27.4)	216 (20.6)		
Positive for another pathogen		165 (22.4)	19 (21.6)	23 (21.3)	25 (22.1)	232 (22.2)		
Negative		429 (58.1)	44 (50.0)	69 (63.9)	57 (50.4)	599 (57.2)		
Source control	3539						39.9, < 0.001	
Yes		655 (24.1)	68 (29.4)	43 (15.3)	102 (32.8)	868 (24.5)		D + , C-
No		1260 (46.4)	97 (42.0)	143 (50.9)	154 (49.5)	1654 (46.7)		
Not applicable		801 (29.5)	66 (28.6)	95 (33.8)	55 (17.7)	1017 (28.7)		D-
Appropriate empirical therapy	3457	1939 (71.3)	154 (66.4)	222 (78.7)	214 (68.8)	2529 (71.3)	20.3, < 0.001	
Total days of active therapy	3393						21.6, 0.001	
<7 days		434 (16.7)	18 (8.0)	53 (19.4)	40 (13.6)	545 (16.1)		B-
7-10 days		907 (34.9)	75 (33.2)	77 (28.2)	100 (34.0)	1159 (34.2)		
>10 days		1259 (48.4)	133 (58.8)	143 (52.4)	154 (52.4)	1689 (49.8)		
Duration of appropriate therapy (days), median (IQR)	1823	11 (7-15)	13 (10-18)	12 (7-16)	12 (8-16)	11 (7-15)	21.2, < 0.001°	B > A
**Outcome**								
Persistent BSI	1517	59 (5.0)	9 (9.5)	9 (7.9)	12 (9.9)	89 (5.9)	8.4; 0.038	None
Clinical cure	3544	2103 (77.3)	185 (79.7)	214 (75.9)	219 (70.4)	2721 (76.8)	8.8, 0.032	D-
30-day mortality	3541	363 (13.4)	19 (8.2)	42 (14.9)	62 (19.9)	486 (13.7)	16.8, 0.001	D + , B-
90-day mortality	3540	498 (18.3)	29 (12.5)	52 (18.4)	91 (29.3)	670 (18.9)	28.5, < 0.001	D + , B-
Relapse_BSI	3497	192 (7.2)	43 (18.8)	33 (11.8)	22 (7.1)	290 (8.3)	42.7, < 0.001	B + , C + , A-
Length of hospital stay (days), median (IQR)	3543	17 (9-34)	28 (14-55)	33 (23-45)	22 (13-37)	19 (10-37)	170.0, < 0.001°	C > A B D, B > A D, D > A

Abbreviations: N = numbers; nm-IC = non major immunocompromised condition; SOT = solid organ transplantation; HM = haematological malignancies; mSC = metastatic solid cancer; IQR = interquartile range; SD = standard deviation;BSI = bloodstream infection; ICU = intensive care unit; CPE = carbapenem resistant Enterobacteraes; SOFA = sequential organ failure assessment; UTI = urinary tract infection; CVC = central venous catheter; ESCR = extended spectrum cephalosporin resistance; FQR = fluoroquinolone resistance; BL/BLI R = beta-lactam/beta-lactamase inhibitors resistance; Carbapenem-R = carbapenem resistance; DTT = difficult to treat resistance; FUBC = follow-up bloodcultures;

* Chi-square test except for: ^ (ANOVA with Sidàk post-hoc test) ° (Kruskal-Wallis test with Holm post-hoc comparisons).

Regarding management, FU-BCs were performed in 22.5% of patients and were more frequent in SOT and HM patients than in the other groups (SOT: 29.7%; HM: 29.4%; mSC: 26.7%; nm-IC: 20.6%, p < 0.001). Source control was performed in 24.5% of GN-BSI. Empiric therapy was appropriate in 71.3% of patients, more frequently in HM group (p < 0.001). Patients received a median treatment course of 11 days (IQR 7–15), with SOT patients receiving longer treatment course compared to other populations (SOT:13 [10–18] days; HM: 12 [7–16] days; mSC:12 [8–16] days; nm-IC: 11 [7–15] days, p < 0.001).

All-cause 30-day mortality was significantly lower in SOT patients, and higher in mSC (SOT 8.2%; nm-IC 13.4%; HM 14.9%; mSC 19.9%, p < 0.001). Kaplan Meier survival analysis curves are shown in S1 Fig. The best fitting model for 30-days mortality in the overall population was a probit with 4 splines (Table 2). Performance of FUBC was associated with a lower risk of death (HR: 0.345, 95%IC: 0.218–0.546, p < 0.001). Factors associated with a higher mortality were age (HR: 1.007, 95% IC: 1.002–1.012, p = 0.005), Charlson Comorbidity Index (CCI) (HR: 1.070, 95% IC: 1.043–1.098, p < 0.001), SOFA score (HR: 1.113, 95% IC: 1.087–1.139, p < 0.001), septic shock (HR: 1.463, 95% IC: 1.200–1.785, p < 0.001), CR profile (HR: 1.387, 95% IC:1.172–1.643, p < 0.001), NFGN etiology (HR: 1.252, 95%IC: 1.034–1.515, p = 0.022); conversely, UTI as source of infection (HR: 0.680, 95% IC:0.562–0.823, p < 0.001) and source control execution (HR: 0.789, 95% IC: 0.666–0.934, p = 0.006) were protective factors. Within subpopulations, FUBC confirmed its association with a lower mortality risk among nm-IC (HR = 0.317, 95% CI 0.178–0.563, p < 0.001), mSC (HR = 0.198, 95% CI 0.058–0.673, p = 0.010) and, not reaching statistical significance, SOT (HR = 0.342, 95% CI 0.092–1.272, p = 0.110), while in the HM subpopulation it was unrelated with mortality (HR = 1.024, 95% CI: 0.248–4.228, p = 0.974) (S4–S7 Tables). The non-proportionality of FUBC related hazard over time was particularly evident in mSC and nm-IC patients (S2 Fig).

**Table 2:** Multivariate survival analysis of 30-day mortality (all patients, n = 3094).

Variable	HR	95% CI	p
FUBC			
Not performed	Ref.	Ref.	Ref.
Performed	0.345	0.218-0.546	<0.001
Age	1.007	1.002-1.012	**0.005**
Males	0.893	0.787-1.014	0.080
CCI	1.070	1.043-1.098	**<0.001**
SOFA score	1.113	1.087-1.139	**<0.001**
Septic shock	1.463	1.200-1.785	**<0.001**
Carbapenem resistance	1.387	1.172-1.643	**<0.001**
Source of BSI			
Primary	Ref.	Ref.	Ref.
Lung	1.185	0.944-1.486	0.143
IAI	0.872	0.722-1.053	0.154
UTI	0.680	0.562-0.823	**<0.001**
CVC	1.113	0.826-1.501	0.481
other	1.099	0.843-1.434	0.485
Source control			
Not performed	Ref.	Ref.	Ref.
Performed	0.789	0.666-0.934	**0.006**
Not applicable	0.929	0.780-1.106	0.409
Appropriate empirical therapy	1.034	0.895-1.193	0.653
Active antibiotic therapy	0.784	0.590-1.042	0.093
Aetiology (NF-GNR)	1.252	1.034-1.515	**0.022**
Parameters of the survival curve			
Spline 1	12.53	7.42-21.15	<0.001
Spline 2	5.58	2.87-10.83	<0.001
Spline 3	0.21	0.08-0.55	0.001
Spline 4	1.74	0.84-3.60	0.137
Spline of FUBC	1.43	1.25-1.64	<0.001

Abbreviations: HR = hazard ratio; CI = confidence interval; CCI = Charlson comorbidity index; SOFA = sequential organ failure assessment; BSI = bloodstream infection; IAI = intra-abdominal infection; UTI = urinary tract infection; CVC = central venous catheter; FUBC = follow up blood cultures; NF-GNR = Non fermentative Gram negative rods.

**Fig 2:**
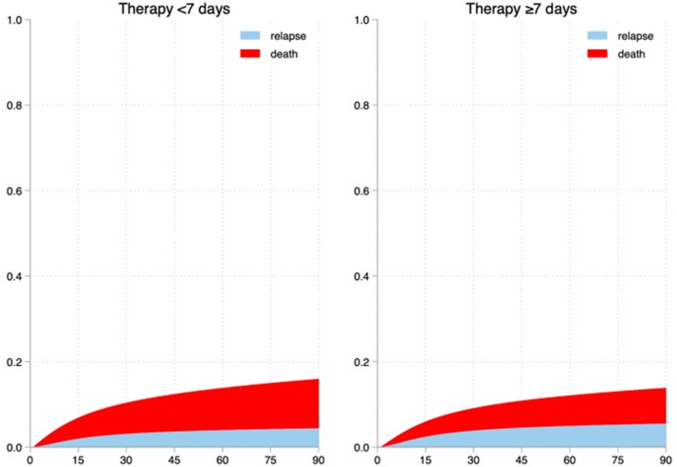
Stacked cumulative incidence of 90-days relapse and death in patients with different duration of the antibiotic course – all patients. **Legend:** Comparison of the cumulative risks of death and relapse among patients stratified by the duration of therapy. Patients receiving less than 7 days of therapy exhibited a higher risk of death compared to relapse.

**Fig 3:**
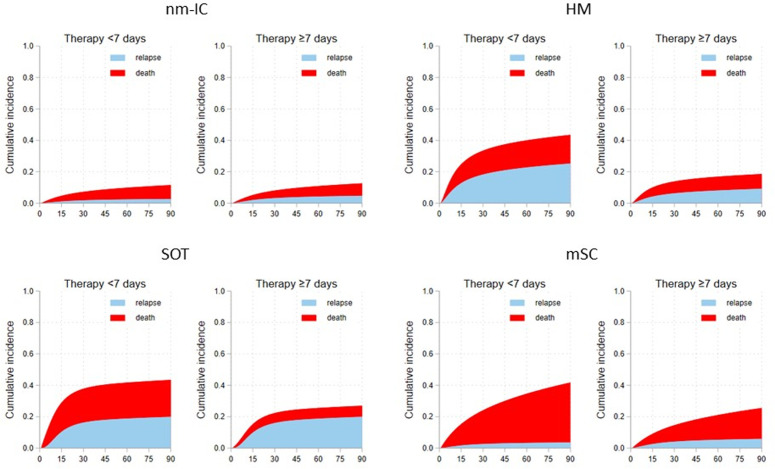
Stacked cumulative incidence of 90-days relapse and death in patients with different duration of the antibiotic course by subpopulation. **Legend:** Cumulative incidence functions for death and relapse for patients with SOT, HM, and mSC, stratified by treatment duration (<7 vs ≥ 7 days). SOT and HM patients exhibited a higher risk of relapse regardless of treatment length, while mSC patients showed a consistently higher risk of death. A ≥ 7-day therapy was associated with reduced mortality in SOT and HM subgroups. nm-IC = non major immunocompromised condition; SOT = solid organ transplantation; HM = haematological malignancies; mSC = metastatic solid cancer.

Rate of 90-day relapse was overall 8.3% and was higher in SOT and HM (SOT: 18.8%; HM: 11.8%; nmIC: 7.2%; mSC: 7.1%, p < 0.001). At multivariable analysis, CR (HR = 2.021, 95% CI:1.597–2.557,p < 0.001) and NFR (HR = 1.541, 95% CI:1.166–2.038, p = 0.002) were the strongest risk factors for relapse, while treatment duration was not significantly associated with increased relapse risk (Table 3). However, as shown in Fig 2, the risk of death was higher than the risk of relapse in patients with less than 7 days of therapy. Among subpopulations, SOT patients and HM patients had the higher risk of relapse regardless of treatment duration; mSC patients had an extremely higher risk of death than relapse regardless of treatment duration, while SOT and HM patients displayed a reduced risk of death when therapy lasted at least 7 days, data as shown in S8–S11 Tables and Fig 3.

**Table 3:** Multivariate survival analysis of 90-day relapse or death (all patients, n = 2943).

Variable	HR	95% CI	p
Relapse	0.039	0.023-0.065	**<0.001**
Death	0.112	0.078-0.160	**<0.001**
Therapy for ≥7 days (relapse)	1.233	0.754-2.014	**0.403**
Therapy for ≥7 days (death)	0.714	0.525-0.972	**0.032**
Age (years)	1.002	0.995-1.009	**0.561**
Males	0.838	0.696-1.008	**0.060**
CCI	1.124	1.087-1.162	**<0.001**
SOFA score	1.123	1.087-1.161	<0.001
Septic shock	1.518	1.110-2.077	**0.009**
Carbapenem resistance	2.021	1.597-2.557	**<0.001**
Source of BSI			
Primary	Ref.	Ref.	Ref.
Lung	0.981	0.690-1.396	0.916
IAI	0.763	0.574-1.013	0.062
UTI	0.686	0.524-0.897	**0.006**
CVC	0.702	0.444-1.112	0.132
other	1.178	0.804-1.728	0.401
Source control			
Not performed	Ref.	Ref.	Ref.
Performed	1.266	0.997-1.607	0.053
Not applicable	1.312	1.039-1.657	**0.023**
Aetiology (NF-GNR)	1.541	1.166-2.038	**0.002**
Parameters of the survival curve			
Spline 1 of relapse survival function	2,03	1.82-2.27	<0.001
Spline 2 of relapse survival function	1.24	1.17-1.31	<0.001
Spline 1 of death survival function	1.65	1.56-1.74	<0.001
Spline 2 of death survival function	1.07	1.04-1.10	<0.001

Abbreviations: HR = hazard ratio; CI = confidence interval; CCI = Charlson comorbidity index; SOFA = sequential organ failure assessment; BSI = bloodstream infection; IAI = intra-abdominal infection; UTI = urinary tract infection; CVC = central venous catheter; FUBC = follow up blood cultures; NF-GNR = Non fermentative Gram negative rods.

## Discussion

We analysed a large cohort of patients with GN BSI focusing on three major groups of IC hosts as SOT recipients, patients with haematological malignancy (HM) and those with metastatic solid cancer (mSC). We observed peculiar differences regarding clinical and microbiological characteristics, and outcome between these populations and non-major immunocompromised study population. Specifically, we observed the highest 30-day mortality rates in the mSC and the lowest in the SOT group. Conversely, the 90-day relapse rate was higher in the SOT group compared to all other populations. Furthermore, we observed that FUBC execution was associated with a lower risk of death in overall population, while its impact was uncertain in SOT and absent in HM. Finally, treatment duration was not associated with a higher risk of relapse, but SOT and HM patients displayed a reduced risk of death when therapy lasted at least 7 days.

Lower mortality rates in SOT recipients with sepsis and/or bacteraemia compared with non-SOT patients have been reported suggesting a protective role of immunosuppressive therapy and/or a more aggressive management in such high-risk setting [3–15]. Conversely, Eichenberger and colleagues recently reported that there was no difference in mortality between SOT and immunocompetent patients with GN-BSI [16]. However, none of these studies provided a detailed description of comparator groups, as in our study. Here, we confirmed that mortality is lower in SOT than in the other groups, but it is worth remarking younger age and lower rate of comorbidities observed in SOT, mainly when compared with mSC and nm-IC patients. Conversely, SOT had the highest risk of relapse. Unfortunately, studies assessing the association between the net state of immunosuppression and outcome of BSI in SOT recipients are not available yet. Otherwise, as for the management, FU-BCs were almost protective against 30-day mortality as well as ≥7 days of treatment duration was associated with reduced 90-day mortality risk.

In patients with HM, a higher rate of fluoroquinolone (FQ) resistance and non-fermenting pathogens were observed compared with other groups. The first is a common place in centres still using FQ prophylaxis [17,18]. In this setting both mortality and relapse rates were higher compared with the other groups, although only for relapse difference was statistically significant. Compared to our cohort, lower mortality rates were reported in a large multicenter Spanish study, ranging from 11% in allogeneic to 5% in autologous HSCT patients [19]. However, the authors reported the outcomes of HSCT patients with BSI due to both Gram-negative and Gram-positive bacteria. In our population, higher SOFA and BSI due to a non-fermenting GNB were associated with increased risk of death, appropriate empiric therapy was protective, while FU-BC was not associated with improved outcome in HM patients (S6 Table). Further, at least 7 days of treatment was not associated with lower relpase but with a reduced 90-day mortality risk. Similarly, Puerta-Alcalde and collegues [20], reported *P. aeruginosa* aetiology and inappropriate empiric antibiotic therapy for GN rods as independent risk factors for mortality in HSCT patients with BSI. In addition, a recent study assessing impact of treatment duration on 30-day recurrence or mortality showed that 7–11 days was not inferior to 12–21 days in 434 haematological patients with *P. aeruginosa* bacteraemia [21]. In our cohort, the median treatment duration of GN-BSI in HM patients was 12 (IQR 7–16) days, also in patients with BSI due to *P. aeruginosa* [12 (7–16) days].

Patients with mSC showed the highest mortality rate (19.9%). This finding is in line with previous evidence, although not all specifically focus on GN etiology, with mortality rates ranging from 13% to 36% [18,22]. The highest mortality rate in our population could be explained also by older age and high rate of comorbidities, while relapse was not more frequent than in other groups. Both FU-BCs and source control were associated with improved 30-day outcome, while treatment duration was not associated with 90-day relapse or mortality risk.

Patients with non-major immunocompromised condition showed a trend toward lower mortality and relapse rates compared with the other groups. FU-BCs were strongly protective, while there was no difference between shorter and longer treatment duration in terms of 90-day relapse or mortality risk. The role of FU-BC is highly debated as randomized controlled trial (RCT) is not available yet [23]. Despite this, two recent metanalysis showed a favourable impact of FU-BC on outcome of patients with GN-BSI [24–25], a recent large population-based study showed no difference in mortality in patients receiving or not FUBC (10.1% vs 8.9%, respectively) [26]. As for treatment duration, the findings from this group confirm what already observed in both observational and RCTs showing non-inferiority of shorter treatment duration in patients with GN-BSI in terms of relapse and/or mortality [27–29]. However, the main limitations of such studies were low representation of immunocompromised patients. In this retrospective analysis on three main groups of immunocompromised patients, surprisingly we did not observe an impact of treatment duration on 90-day relapse risk but a reduced risk of 90-day mortality in SOT and HM patients treated at least 7 days. Further multicentre studies are needed to confirm these findings.

There are several limitations in this study. We analysed a large cohort of patients, but the single-center design could limit the generalizability of our results. We focused on three specific types of IC condition, for which the increased risk of infectious complications is well-defined, considering all the other patients as not having a major IC status. However, patients included in this group could have been affected by other types and degrees of IC. We tried to take into account this occurrence by adjusting multivariable analysis for the Charlson index score. As for solid cancer, we considered only patients with a metastatic solid cancer for their higher fragility, but also patients with localized solid tumor could present with several degrees of immunosuppression. Finally, despite the adjustment for potential confounding variables, the use of FU-BC as time dependent variable, and considering patients since the end of therapy for the analysis of treatment duration, selection and immortal time bias could not be fully eliminated.

In conclusion, our data suggest that the characteristics and outcome of GN-BSI is different between the three specific IC categories of SOT recipients, patients with HM and those with metastatic solid cancer. Management of GN-BSI should be targeted according with the type of IC patient. Large interventional studies are needed to investigate the impact of specific procedures, as FU-BC, as well as the optimal treatment duration in each IC group.

## Supporting information

S1 File Variables and definitions. (DOCX)

S1 Table SOT patients characteristics. (DOCX)

S2 Table Hematologic malignancy patients characteristics. (DOCX)

S3 Table Characteristics of patients with metastatic solid tumor. (DOCX)

S4 Table Multivariable survival analysis of 30-day mortality in nm-IC population. (DOCX)

S5 Table Multivariable survival analysis of 30-day mortality in SOT population. (DOCX)

S6 Table Multivariable survival analysis of 30-day mortality in HM population. (DOCX)

S7 Table Multivariable survival analysis of 30-day mortality in mSC population. (DOCX)

S8 Table Multivariable survival analysis of 90-day relapse or death in nm-IC population. (DOCX)

S9 Table Multivariable survival analysis of 90-day relapse or death in SOT population. (DOCX)

S10 Table Multivariable survival analysis of 90-day relapse or death in HM population. (DOCX)

S11 Table Multivariable survival analysis of 90-day relapse or death in mSC population. (DOCX)

S1 Fig 30-day overall survival by IC subpopulation. IC = immunocompromised condition; nm-IC = non major immunocompromised condition; SOT = solid organ transplantation; HM = haematological malignancies; mSC = metastatic solid cancer.
(JPG)

S2 Fig Model-estimated survival function regarding 30-day mortality for FUBC execution vs not execution. FUBC = follow up bloodcultures; nm-IC = non major immunocompromised condition; SOT = solid organ transplantation; HM = haematological malignancies; mSC = metastatic solid cancer.
(JPG)
