# Supplementary material for: Epidemiological characteristics and management of Gram-negative bacteraemia in different immunocompromised hosts: Observational single-center study
Source: PLoS One. 2025 Jul 7;20(7):e0327535. doi: 10.1371/journal.pone.0327535 (PMC12233224; doi:10.1371/journal.pone.0327535)
Supplement: S1 File — (DOCX) [file pone.0327535.s001.docx]

**S1 File.** **Supplementary materials and methods**

**METHODS**

Variables and Definitions

For each patients group, the following variables were collected:

- Solid organ transplant (SOT) recipients: type of organ transplanted (heart, lung, liver, kidney), maintenance immunosuppressive therapy at time of index BSI, functional status of the graft at the time of index BC, if present induction immunosuppressive therapy in the last 6 months before GN-BSI.
- Hematologic malignancy (HM) patients: type of HM including acute myeloid leukemia (AML), acute lymphoblastic leukemia (ALL), chronic lymphoblastic leukemia (CLL), multiple myeloma (MM), aplastic anemia (AA), chronic myeloid leukemia (CML), myelodisplastic syndrome (MS), non-Hodgkin lymphoma (NHL), Hodgkin lymphoma (HL). Patients with haematopoietic stem cell transplantation (HSCT) were also included. Data on chemotherapy regimens were collected.
- metastatic solid cancer (mSC) patients: type of primary neoplasia, surgery, hormone therapy, immunotherapy, radiotherapy and chemotherapy carried out or ongoing at the time of index BSI.

Variables collected for all patients included age, sex, Charlson comorbidity index (CCI) (1), BSI classified as hospital or community acquired according to the Friedman criteria (2), clinical severity at infection onset defined according to SOFA and septic shock criteria (3), inpatient ward (i.e. internal medicine, ICU, Surgery, Emergency department) at the time of index BSI, A previous GN-BSI was defined if a GN-BSI (due to different GN microorganism) occurred more than 90 days before the index BSI.

BSI sources were established according to US Centers for Disease Control and Prevention (CDC) criteria (4). BSI was defined as “primary” in case of unidentified source of infection. Isolates were summarized in *Enterobacterales* (*Klebsiella spp, Escherichia coli, Enterobacter spp, Citrobacter spp, Serratia spp, Morganella spp and Proteus spp*) and Non fermentative Gram-negative (NFGN) (*Pseudomonas spp, Acinetobacter spp, Stenotrophomonas spp*). Strains were further categorized according to their resistance profile as carbapenem resistant (CR), extended-spectrum cephalosporin resistant (ECR), β-lactam/β-lactamase inhibitor resistant (BL/BLIR) and fluroquinolone resistant (FQR) according to CDC criteria (5); the difficult to treat resistant (DTR) category was also assessed (6).

FUBCs were defined as BCs drawn between 48 hours and 7 days after index BCs. Results of FUBCs were classified as positive for the same pathogen; positive for a different pathogen from that of index BCs; and negative. Attained source control was defined as the removal of the infection source within 7 days of index BCs, including the performance of non-surgical or surgical procedures to treat an obstructive focus or abscess at any site including, among others, the urinary tract, biliary tract and surgical site, and the removal of any device deemed as the source of BSI.

Empirical antibiotic therapy was defined as the administration of an antimicrobial before receiving the susceptibility report of BSI isolate. It was considered appropriate if at least one antibiotic among those administered had in vitro activity according to susceptibility results. Duration of active antibiotic therapy was defined as the number of consecutive days during which the patient received an appropriate antibiotic regimen.

References:

1. Charlson ME, Pompei P, Ales KL, MacKenzie CR. A new method of classifying prognostic comorbidity in longitudinal studies: Development and validation. J Chronic Dis.1987;40(5):373–83.

2. Friedman ND. Health Care–Associated Bloodstream Infections in Adults: A Reason To Change the Accepted Definition of Community-Acquired Infections. Ann Intern Med.2002;137(10):791.

3. Singer M, Deutschman CS, Seymour CW, Shankar-Hari M, Annane D, Bauer M, et al. The Third International Consensus Definitions for Sepsis and Septic Shock (Sepsis-3). JAMA. 2016;315(8):801.

4. Horan TC, Andrus M, Dudeck MA. CDC/NHSN surveillance definition of health care–associated infection and criteria for specific types of infections in the acute care setting. Am J Infect Control. 2008;36(5):309–32.

5. Magiorakos AP, Srinivasan A, Carey RB, Carmeli Y, Falagas ME, Giske CG, et al. Multidrug-resistant, extensively drug-resistant and pandrug-resistant bacteria: an international expert proposal for interim standard definitions for acquired resistance. Clin Microbiol Infect. 2012;18(3):268–81.

6. Kadri SS, Adjemian J, Lai YL, Spaulding AB, Ricotta E, Prevots DR, et al. Difficult-to-Treat Resistance in Gram-negative Bacteremia at 173 US Hospitals: Retrospective Cohort Analysis of Prevalence, Predictors, and Outcome of Resistance to All First-line Agents. Clin Infect Dis Off Publ Infect Dis Soc Am. 2018;67(12):1803–14.
