# Supplementary material for: Epidemiological characteristics and management of Gram-negative bacteraemia in different immunocompromised hosts: Observational single-center study
Source: PLoS One. 2025 Jul 7;20(7):e0327535. doi: 10.1371/journal.pone.0327535 (PMC12233224; doi:10.1371/journal.pone.0327535)
Supplement: S1 Table — (DOCX) [file pone.0327535.s002.docx]

**S1 Table. SOT patients characteristics**

|  | **N 232 (%)** |
| --- | --- |
| Graft type |  |
| *Heart* | 14 (6.0) |
| *Lung* | 7 (3.0) |
| *Liver* | 130 (56.0) |
| *Kidney* | 87 (37.5) |
| BSI donor derived | 5 (2.2) |
| Days from SOT to BSI diagnosis (median, IQR) | 164 (18-2063) |
| Leucopenia at BSI diagnosis |  |
| *Absolute leucopenie (WBC <1000/ml)* | 4 (1.7) |
| *Relative leucopenia (WBC < 3500/ml)* | 29 (12.5) |
| Induction in the last 6 months | 114 (49.1) |
| *Steroids* | 104 (91.2) |
| *Antylimpphocyte globulin* | 27 (23.7) |
| *Basiliximab/daclizumab* | 30 (26.3) |
| *Alemtuzumab* | 0 (0.0) |
| *Rituximab* | 1 (0.9) |
| *Betalacept* | 0 (0) |
| Maintenance regime at time of BSI |  |
| *CNI* | 212 (91.4) |
| *-Cyclosporine* | 25 (10.8) |
| *-Tacrolimus* | 187 (80.6) |
| *Mycophenolate* | 62 (26.7) |
| *Azathioprine* | 5 (2.1) |
| *Sirolimus* | 3 (1.3) |
| *Everolimus* | 6 (2.6) |
| *Steroids* | 180 (77.6) |
| CMV infection/disease in the last 30 days | 14 (6.0) |
| *Infection* | 11 (78.6) |
| *Disease* | 3 (21.4) |
| Graft function at time of GN-BSI diagnosis |  |
| *Good* | 108 (47.2) |
| *Impaired* | 93 (40.6) |
| *Failure* | 22 (9.6) |
| *Rejection* | 6 (2.8) |

**Abbreviations**:SOT: solid organ transplant, BSI: bloodstream infection, IQR: interquartile range, CMV: cytomegalovirus, CNI: Calcineurin inhibitors , GN-BSI: Gram-negative BSI, SOT: solid organ transplant, WBC: white blood cells.
