# Supplementary material for: Epidemiological characteristics and management of Gram-negative bacteraemia in different immunocompromised hosts: Observational single-center study
Source: PLoS One. 2025 Jul 7;20(7):e0327535. doi: 10.1371/journal.pone.0327535 (PMC12233224; doi:10.1371/journal.pone.0327535)
Supplement: S2 Table — (DOCX) [file pone.0327535.s003.docx]

**S2 Table - Hematologic malignancy patients characteristics**

|  | **N 282 (%)** |
| --- | --- |
| Haematological disease |  |
| *AML* | 126 (44.7) |
| *HL* | 8 (2.8) |
| *ALL* | 34 (12.1) |
| *MM* | 33 (11.7) |
| *CLL* | 4 (1.4) |
| *AA* | 0 (0) |
| *ML* | 6 (2.1) |
| *NHL* | 58 (20.6) |
| *Other* | 2 (0.7) |
| HSCT | 100 (35.5) |
| Days from HSCT to BSI diagnosis (median, IQR) | 7.5 (5-31) |
| Reason for admission |  |
| *Induction chemotherapy* | 65 (23.0) |
| *Consolidation chemoteraphy* | 36 (12.8) |
| *Salvage Chemoterapy* | 68 (24.1) |
| *HSCT* | 86 (30.5) |
| *Other* | 27 (9.6) |
| Prior lines of therapy |  |
| *First line* | 215 (76.2) |
| *1* | 18 (6.4) |
| *2* | 22 (7.8) |
| *3* | 16 (5.7) |
| *4* | 5 (1.8) |
| Neutropenia (Neutrophil count <500/mmc) | 234 (83.0) |
| Infection prevention strategy during neutropenia |  |
| *None* | 36 (15.5) |
| *Levofloxacin* | 170 (73.0) |
| *Other* | 27 (11.6) |
| CMV infection/disease in the last 30 days | 10 (3.5) |
| *Infection* | *8 (80)* |
| *Disease* | *2 (20)* |
| Acute GVHD at BSI diagnosis | 12 (4.3) |
| *Skin* | 9 (75.0) |
| *Liver* | 2 (16.0) |
| *GI tract* | 8 (66.6) |
| *Lungs* | 1 (8.3) |
| Chronic GVHD at BSI diagnosis | 2 (0.7) |
| *Skin* | 2 (100) |
| *Liver* | 1 (50) |

**Abbreviations**: AA: aplastic anemia; ALL: acute lymphoblastic leukemia; AML: acute myeloid leukemia; BSI: bloodstream infections; CLL: chronic lymphoblastic leukemia; CML: chronic myeloid leukemia; GI : gastro-intestinal; GVHD: graft-versus-host disease; HL: Hodgkin lymphoma; HSCT: haematopoietic stem cell transplantation; MM: multiple myeloma; NHL: non-Hodgkin lymphoma, CMV: cytomegalovirus, IQR: interquartile range.
