# Supplementary material for: Epidemiological characteristics and management of Gram-negative bacteraemia in different immunocompromised hosts: Observational single-center study
Source: PLoS One. 2025 Jul 7;20(7):e0327535. doi: 10.1371/journal.pone.0327535 (PMC12233224; doi:10.1371/journal.pone.0327535)
Supplement: S3 Table — (DOCX) [file pone.0327535.s004.docx]

**S3 Table – Characteristics of patients with metastatic solid tumor**

|  | **N:311 (%)** |
| --- | --- |
| Type of cancer |  |
| *Lung* | 12 (3.9) |
| *Breast* | 9 (2.9) |
| *Colorectal* | 63 (20.3) |
| *Prostate* | 18 (5.8) |
| *Kidney* | 3 (1.0) |
| *Bladder* | 15 (4.8) |
| *Head-neck* | 3 (1.0) |
| *Melanoma* | 2 (0.6) |
| *Sarcoma* | 5 (1.6) |
| *Ovary* | 18 (5.8) |
| *Pancreas* | 56 (18.0) |
| *Stomach* | 14 (4.5) |
| *Esophagus* | 2 (0.6) |
| *Liver* | 7 (2.3) |
| *Biliary Tract* | 41 (13.2) |
| *Other gynecological tumors* | 15 (4.8) |
| *Other* | 25 (8.0) |
| Surgery prior BSI | 123 (39.5) |
| Radiotherapy in the last 3 months | 19 (6.1) |
| Oncological therapy ongoing | 64 (20.6) |
| *Chemotherapy* | 49 (76.7) |
| *Hormonal therapy* | 13 (20.3) |
| *TKI therapy* | 1 (1.5) |
| *Immunotherapy* | 4 (6.3) |
| Days from last chemotherapy to BSI diagnosis (median, IQR) | 76 (29-214) |
| Neoadjuvant | 2 (0.6) |
| Adjuvant | 11 (3.5) |
| Subsequent lines | 5 (1.6) |

**Abbreviations**: BSI: bloodstream infections; TKI: Tyrosine Kinase Inhibitor, IQR: interquartile range.
