# Supplementary material for: Epidemiological characteristics and management of Gram-negative bacteraemia in different immunocompromised hosts: Observational single-center study
Source: PLoS One. 2025 Jul 7;20(7):e0327535. doi: 10.1371/journal.pone.0327535 (PMC12233224; doi:10.1371/journal.pone.0327535)
Supplement: S4 Table — (DOCX) [file pone.0327535.s005.docx]

**S 4 Table. Survival analysis of 30-day mortality in nm-IC population (n=2346)**

| **Variable** | **HR** | **95% CI** | **p-value** |
| --- | --- | --- | --- |
|  |  |  |  |
| FUBC |  |  |  |
| Not performed | Ref. | Ref. | Ref. |
| Performed | 0.317 | 0.178-0.563 | **<0.001** |
| Age | 1.013 | 1.006-1.020 | **<0.001** |
| Males | 0.908 | 0.781-1.055 | 0.206 |
| CCI | 1.061 | 1.025-1.099 | **0.001** |
| SOFA score | 1.104 | 1.074-1.134 | **<0.001** |
| Aetiology (GN-GNR) | 1.307 | 1.041-1.641 | **0.021** |
| Septic Shock | 1.608 | 1.289-2.006 | **<0.001** |
| Carbapenem resistance | 1.461 | 1.192-1.791 | **<0.001** |
| Appropriate empirical therapy | 1.110 | 0.938-1.312 | 0.224 |
| Active antibiotic therapy | 0.860 | 0.615-1.203 | 0.379 |
|  |  |  |  |
| Source of BSI |  |  |  |
| Primary | Ref. | Ref. | Ref. |
| Lung | 1.238 | 0.952-1.610 | 0.111 |
| IAI | 0.906 | 0.718-1.141 | 0.401 |
| UTI | 0.661 | 0.527-0.829 | **<0.001** |
| CVC | 0.991 | 0.685-1.433 | 0.961 |
| Other | 1.171 | 0.844-1.625 | 0.345 |
| Source control |  |  |  |
| Not performed | Ref. | Ref. | Ref. |
| Performed | 0.848 | 0.696-1.034 | 0.103 |
| Not applicable | 0.929 | 0.758-1.140 | 0.482 |
| Parameters of the survival curve |  |  |  |
| Spline 1 | 6.346 | 4.187-9.618 | <0.001 |
| Spline 2 | 2.067 | 1.496-2.857 | <0.001 |
| Spline 3 | 0.593 | 0.406-0.866 | 0.007 |
| Spline of_FUBC | 1.481 | 1.247-1.759 | <0.001 |
| _cons | 0.004 | 0.002-0.010 | <0.001 |
| Abbreviations: HR= hazard ratio; CI=confidence interval; FUBC= follow up blood cultures CCI=Charlson comorbidity index; SOFA=sequential organ failure assessment; BSI= bloodstream infection; IAI=intra-abdominal infection; UTI= urinary tract infection; CVC=central venous catheter; NF-GNR= Non fermentative Gram negative rods. | | | |
