# Supplementary material for: Epidemiological characteristics and management of Gram-negative bacteraemia in different immunocompromised hosts: Observational single-center study
Source: PLoS One. 2025 Jul 7;20(7):e0327535. doi: 10.1371/journal.pone.0327535 (PMC12233224; doi:10.1371/journal.pone.0327535)
Supplement: S5 Table — (DOCX) [file pone.0327535.s006.docx]

**S 5 Table. Multivariable survival analysis of 30-day mortality in SOT population (n=205)**

|  |  |  |  |
| --- | --- | --- | --- |
| **Variable** | **HR** | **95% CI** | **p-value** |
|  |  |  |  |
| FUBC |  |  |  |
| Not performed | Ref. | Ref. | Ref. |
| Performed | 0.343 | 0.092-1.272 | 0.110 |
| Age | 1.006 | 0.954-1.060 | 0.824 |
| Males | 0.571 | 0.181-1.798 | 0.338 |
| CCI | 1.326 | 0.966-1.820 | **0.081** |
| SOFA | 1.160 | 0.955-1.409 | 0.135 |
| Aetiology (NF-GNR) | 0.171 | 0.014-2.065 | 0.165 |
| Septic shock | 0.750 | 0.104-5.396 | 0.775 |
| Carbapenem resistance | 4.527 | 1.321-15.514 | **0.016** |
| Appropriate empirical therapy | 0.416 | 0.094-1.845 | 0.248 |
| Source of BSI |  |  |  |
| Primary | Ref. | Ref. | Ref. |
| Lung | 2.173 | 0.333-14.191 | 0.417 |
| IAI | 1.306 | 0.249-6.846 | 0.752 |
| UTI | 0.261 | 0.025-2.736 | 0.263 |
| Other | 2.196 | 0.171-28.247 | 0.546 |
| CVC | 11.273 | 1.039-122.328 | **0.046** |
| Source control |  |  |  |
| Not performed | Ref. | Ref. | Ref. |
| Performed | 0.660 | 0.117-3.736 | 0.638 |
| Not applicable | 3.546 | 0.634-19.836 | 0.150 |
| Constable | 0.003 | 0.000-0.019 | 0.000 |
| Abbreviations: HR= hazard ratio; CI=confidence interval; FUBC= follow up blood cultures CCI=Charlson comorbidity index; SOFA=sequential organ failure assessment; BSI= bloodstream infection; IAI=intra-abdominal infection; UTI= urinary tract infection; CVC=central venous catheter; NF-GNR= Non fermentative Gram negative rods. | | | |
