# Supplementary material for: Epidemiological characteristics and management of Gram-negative bacteraemia in different immunocompromised hosts: Observational single-center study
Source: PLoS One. 2025 Jul 7;20(7):e0327535. doi: 10.1371/journal.pone.0327535 (PMC12233224; doi:10.1371/journal.pone.0327535)
Supplement: S6 Table — (DOCX) [file pone.0327535.s007.docx]

**S 6 Table. Multivariable survival analysis of 30-day mortality in HM population (n=258)**

| **Variable** | **HR** | **95% CI** | **p-value** |
| --- | --- | --- | --- |
| FUBC |  |  |  |
| Not performed | Ref. | Ref. | Ref. |
| Performed | 1.024 | 0.248-4.228 | 0.974 |
| Age | 1.000 | 0.981-1.021 | 0.964 |
| Males | 0.729 | 0.457-1.161 | 0.183 |
| CCI | 1.103 | 0.970-1.254 | 0.135 |
| SOFA | 1.318 | 1.151-1.508 | **<0.001** |
| Aetiology (NF-GNR) | 2.693 | 1.264-5.735 | **0.010** |
| Septic Shock | 0.579 | 0.148-2.260 | 0.431 |
| Carbapenem resistance | 0.997 | 0.484-2.055 | 0.994 |
| Appropriate empirical therapy | 0.503 | 0.268-0.944 | **0.033** |
| Active antibiotic therapy | 0.625 | 0.212-1.840 | 0.393 |
| Source of BSI |  |  |  |
| Primary | Ref. | Ref. | Ref. |
| Lung | 0.594 | 0.250-1.413 | 0.239 |
| IAI | 0.945 | 0.458-1.947 | 0.877 |
| UTI | 1.477 | 0.629-3.472 | 0.371 |
| Other | 0.458 | 0.128-1.638 | 0.230 |
| CVC | 1.079 | 0.440-2.643 | 0.868 |
| Source control |  |  |  |
| Not performed | Ref. | Ref. | Ref. |
| Performed | 1.014 | 0.475-2.164 | 0.972 |
| Not applicable | 1.343 | 0.686-2.627 | 0.389 |
| Parameters of the survival curve |  |  |  |
| Spline1 | 2.782 | 1.655-4.676 | 0.000 |
| Spline2 | 1.204 | 0.954-1.521 | 0.118 |
| Spline of FUBC | 1.004 | 0.658-1.532 | 0.986 |
| Constable | 0.025 | 0.005-0.117 | 0.000 |
| Abbreviations: HR= hazard ratio; CI=confidence interval; FUBC= follow up blood cultures CCI=Charlson comorbidity index; SOFA=sequential organ failure assessment; BSI= bloodstream infection; IAI=intra-abdominal infection; UTI= urinary tract infection; CVC=central venous catheter; NF-GNR= Non fermentative Gram negative rods. | | | |
