# Supplementary material for: Epidemiological characteristics and management of Gram-negative bacteraemia in different immunocompromised hosts: Observational single-center study
Source: PLoS One. 2025 Jul 7;20(7):e0327535. doi: 10.1371/journal.pone.0327535 (PMC12233224; doi:10.1371/journal.pone.0327535)
Supplement: S7 Table — (DOCX) [file pone.0327535.s008.docx]

**S 7 Table: Multivariable survival analysis of 30-day mortality in mSC population (n=285)**

|  |  |  |  |
| --- | --- | --- | --- |
| **Variable** | **HR** | **95% CI** | **p-value** |
| FUBC | 0.198 | 0.058-0.673 | **0.010** |
| Age | 1.017 | 0.996-1.039 | 0.118 |
| Males | 1.345 | 0.915-1.979 | 0.132 |
| CCI | 0.901 | 0.799-1.017 | 0.092 |
| SOFA | 1.225 | 1.133-1.325 | **<0.001** |
| NF-GNR | 0.722 | 0.377-1.383 | 0.325 |
| Septic shock | 0.918 | 0.448-1.884 | 0.816 |
| Carbapenem resistance | 1.511 | 0.902-2.532 | 0.117 |
| Appropriate empirical therapy | 1.127 | 0.728-1.746 | 0.592 |
| Active antibiotic therapy | 0.560 | 0.2601.208 | 0.139 |
| Source of BSI |  |  |  |
| Primary | Ref. | Ref. | Ref. |
| Lung | 0.923 | 0.353-2.412 | 0.870 |
| IAI | 0.648 | 0.331-1.269 | 0.206 |
| UTI | 0.879 | 0.431-1.796 | 0.724 |
| Other | 1.297 | 0.558-3.016 | 0.546 |
| CVC | 1.259 | 0.480-3.303 | 0.640 |
| Source control |  |  |  |
| Not performed | Ref. | Ref. | Ref. |
| Performed | 0.575 | 0.360-0.918 | **0.020** |
| Not applicable | 0.707 | 0.388-1.287 | 0.257 |
| Spline 1 | 5.513 | 3.216-9.451 | 0.000 |
| Spline 2 | 2.016 | 1.558-2.607 | 0.000 |
| Spline of FUBC | 1.692 | 1.166-2.456 | 0.006 |
| Constant | 0.016 | 0.003-0.079 | 0.000 |
| Abbreviations: HR= hazard ratio; CI=confidence interval; FUBC= follow up blood cultures CCI=Charlson comorbidity index; SOFA=sequential organ failure assessment; BSI= bloodstream infection; IAI=intra-abdominal infection; UTI= urinary tract infection; CVC=central venous catheter; NF-GNR= Non fermentative Gram negative rods. | | | |
