# Supplementary material for: Epidemiological characteristics and management of Gram-negative bacteraemia in different immunocompromised hosts: Observational single-center study
Source: PLoS One. 2025 Jul 7;20(7):e0327535. doi: 10.1371/journal.pone.0327535 (PMC12233224; doi:10.1371/journal.pone.0327535)
Supplement: S8 Table — (DOCX) [file pone.0327535.s009.docx]

**Supplementary Table 8:** **Multivariable survival analysis of 90-day relapse or death in nm-IC population**

| **Variable** | **HR** | **95% CI** | **p-value** |
| --- | --- | --- | --- |
| Relapse | 0.024 | 0.012-0.049 | **<0.001** |
| Death | 0.085 | 0.054-0.133 | **<0.001** |
| Duration of therapy (relapse) | 1.777 | 0.897-3.522 | 0.099 |
| Duration of therapy (death) | 0.895 | 0.608-1.317 | 0.573 |
| Age | 1.007 | 0.997-1.016 | 0.118 |
| Males | 0.890 | 0.711-1.113 | 0.305 |
| CCI | 1.110 | 1.054-1.168 | **<0.001** |
| SOFA | 1.107 | 1.063-1.152 | **<0.001** |
| NF-GNR | 1.661 | 1.176-2.346 | **0.004** |
| Septic shock | 1.594 | 1.101-2.307 | 0.013 |
| Carbapenem resistance | 1.968 | 1.468-2.638 | **<0.001** |
| Source of BSI |  |  |  |
| Primary | Ref. | Ref. | Ref. |
| Lung | 0.987 | 0.656-1.483 | 0.948 |
| IAI | 0.777 | 0.548-1.102 | 0.157 |
| UTI | 0.599 | 0.436-0.823 | **0.002** |
| Other | 0.987 | 0.598-1.639 | 0.960 |
| CVC | 0.637 | 0.360-1,126 | 0.121 |
| Source control |  |  |  |
| Not performed | Ref. | Ref. | Ref. |
| Performed | 1.416 | 1.058-1.895 | **0.019** |
| Not applicable | 1.518 | 1.150-2.003 | **0.003** |
| RCS relapse 1 | 1.933 | 1.702-2.196 | <0.001 |
| RCS relapse 2 | 1.209 | 1.127-1.296 | <0.001 |
| RCS death 1 | 1.617 | 1.518-1.723 | <0.001 |
| RCS death 2 | 1.068 | 1.033-1.104 | <0.001 |
| Abbreviations: HR= hazard ratio; CI=confidence interval; SOFA=sequential organ failure assessment; BSI= bloodstream infection; IAI=intra-abdominal infection; UTI= urinary tract infection; CVC=central venous catheter; NF-GNR= Non fermentative Gram negative rods. | | | |
