# Supplementary material for: Epidemiological characteristics and management of Gram-negative bacteraemia in different immunocompromised hosts: Observational single-center study
Source: PLoS One. 2025 Jul 7;20(7):e0327535. doi: 10.1371/journal.pone.0327535 (PMC12233224; doi:10.1371/journal.pone.0327535)
Supplement: S9 Table — (DOCX) [file pone.0327535.s010.docx]

**Supplementary Table 9****: Multivariable survival analysis of 90-day relapse or death in SOT population**

| **Variable** | **HR** | **95% CI** | **p-value** |
| --- | --- | --- | --- |
| Relapse | 0.189 | 0.048-0.751 | **0.018** |
| Death | 0.286 | 0.083-0.987 | **0.048** |
| Duration of therapy (relapse) | 0.823 | 0.229-2.952 | 0.765 |
| Duration of therapy (death) | 0.260 | 0.075-0,901 | **0.034** |
| Age | 0.996 | 0.966-1.027 | 0.803 |
| Males | 0.753 | 0.376-1.509 | 0.424 |
| CCI | 1.183 | 1.007-1.390 | **0.041** |
| SOFA | 1.042 | 0.922-1.176 | 0.511 |
| NF-GNR | 0.513 | 0.160-1.641 | 0.260 |
| Septic shock | 3.751 | 1.112-12.650 | **0.033** |
| Carbapenem resistance | 2.176 | 1.075-4.403 | **0.031** |
| Source of BSI |  |  |  |
| Primary | Ref. | Ref. | Ref. |
| Lung | 1.101 | 0.263-4.615 | 0.896 |
| IAI | 0.660 | 0.266-1.638 | 0.370 |
| UTI | 0.759 | 0.297-1.941 | 0.565 |
| Other | 0.592 | 0.118-2.969 | 0.524 |
| CVC | 1.842 | 0.403-8.417 | 0.430 |
| Source control |  |  |  |
| Not performed | Ref. | Ref. | Ref. |
| Performed | 0.633 | 0.268-1.500 | 0.298 |
| Not applicable | 0.849 | 0.364-2.00 | 0.705 |
| Spline relapse 1 | 3.334 | 2.084-5.335 | <0.001 |
| Spline relapse 2 | 1.796 | 1.337-2.413 | <0.001 |
| Spline death 1 | 1.455 | 1.207-1.754 | <0.001 |
| Spline death 2 | 1.179 | 1.036-1.340 | 0.012 |
| Abbreviations: HR= hazard ratio; CI=confidence interval; SOFA=sequential organ failure assessment; BSI= bloodstream infection; IAI=intra-abdominal infection; UTI= urinary tract infection; CVC=central venous catheter; NF-GNR= Non fermentative Gram negative rods. | | | |
