# Supplementary material for: Epidemiological characteristics and management of Gram-negative bacteraemia in different immunocompromised hosts: Observational single-center study
Source: PLoS One. 2025 Jul 7;20(7):e0327535. doi: 10.1371/journal.pone.0327535 (PMC12233224; doi:10.1371/journal.pone.0327535)
Supplement: S10 Table — (DOCX) [file pone.0327535.s011.docx]

**Supplementary Table 10: Multivariable survival analysis of 90-day relapse or death in HM population**

| **Variable** | **HR** | **95% CI** | **p-value** |
| --- | --- | --- | --- |
| Relapse | 0.263 | 0.087-0.800 | **0.019** |
| Death | 0.200 | 0.060-0.670 | **0.009** |
| Duration of therapy (relapse) | 0.300 | 0.100-0.903 | **0.032** |
| Duration of therapy (death) | 0.443 | 0.978-1.426 | 0.172 |
| Age | 1.009 | 0.978-1.041 | 0.560 |
| Males | 0.411 | 0.202-0.836 | **0.014** |
| CCI | 1.215 | 1.037-1.423 | **0.016** |
| SOFA | 1.284 | 1.099-1.500 | **0.002** |
| NF-GNR | 0.983 | 0.394-2.341 | 0.970 |
| Septic shock | 2.106 | 0.380-11.680 | 0.394 |
| Carbapenem resistance | 3.300 | 1.224-8.896 | **0.018** |
| Source of BSI |  |  |  |
| Primary | Ref. | Ref. | Ref. |
| Lung | 0.878 | 0.225-3.433 | 0.852 |
| IAI | 0.380 | 0.078-1.837 | 0.229 |
| UTI | 2.607 | 0.883-7.693 | 0.083 |
| Other | 2.614 | 0.799-8.554 | 0.112 |
| CVC | 2.889 | 0.795-10.492 | 0.107 |
| Source control |  |  |  |
| Not performed | Ref. | Ref. | Ref. |
| Performed | 0.759 | 0.262-2.195 | 0.611 |
| Not applicable | 0.622 | 0.254-1.524 | 0.299 |
| Spline relapse 1 | 2.199 | 1.617-2.990 | <0.001 |
| Spline relapse 2 | 1.221 | 1.043-1.428 | 0.013 |
| Spline death 1 | 1.725 | 1.399-2.128 | <0.001 |
| Spline death 2 | 1.172 | 1,056-1.301 | 0.003 |
| Abbreviations: HR= hazard ratio; CI=confidence interval; SOFA=sequential organ failure assessment; BSI= bloodstream infection; IAI=intra-abdominal infection; UTI= urinary tract infection; CVC=central venous catheter; NF-GNR= Non fermentative Gram negative rods. | | | |
