# Supplementary material for: Epidemiological characteristics and management of Gram-negative bacteraemia in different immunocompromised hosts: Observational single-center study
Source: PLoS One. 2025 Jul 7;20(7):e0327535. doi: 10.1371/journal.pone.0327535 (PMC12233224; doi:10.1371/journal.pone.0327535)
Supplement: S11 Table — (DOCX) [file pone.0327535.s012.docx]

**Supplementary Table 11:** **Multivariable survival analysis of 90-day relapse or death in mSC population**

| **Variable** | **HR** | **95% CI** | **p-value** |
| --- | --- | --- | --- |
| Relapse | 0.034 | 0.004-0.319 | **0.003** |
| Death | 0.383 | 0.111-1.321 | 0.129 |
| Duration of therapy (relapse) | 1.460 | 0.186-11.444 | 0.719 |
| Duration of therapy (death) | 0.456 | 0.222-0.936 | 0.032 |
| Age | 1.997 | 0.982-1.033 | 0.567 |
| Males | 0.956 | 0.563-1.621 | 0.867 |
| CCI | 1.010 | 0.889-1.147 | 0.879 |
| SOFA | 1.173 | 1.060-1.298 | **0.002** |
| NF-GNR | 1.891 | 0.841-4.249 | 0.123 |
| Septic shock | 0.873 | 0.309-2.468 | 0.798 |
| Carbapenem resistance | 2.443 | 1.202-4.966 | **0.014** |
| Source of BSI |  |  |  |
| Primary | Ref. | Ref. | Ref. |
| Lung | 0.744 | 0.200-2.768 | 0.659 |
| IAI | 0.524 | 0.201-1.366 | 0.186 |
| UTI | 0.844 | 0.329-2.165 | 0.724 |
| Other | 1.201 | 0.402-3.587 | 0.743 |
| CVC | 0.182 | 0.033-0.999 | **0.050** |
| Source control |  |  |  |
| Not performed | Ref. | Ref. | Ref. |
| Performed | 1.204 | 0.670-2.162 | 0.535 |
| Not applicable | 1.380 | 0.646-2.945 | 0.406 |
| Spline relapse 1 | 2.298 | 1.442-3.660 | <0.001 |
| Spline relapse 2 | 1.257 | 0.985-1.604 | 0.066 |
| Spline death 1 | 2-033 | 1.693-2.442 | <0.001 |
| Spline death 2 | 1.047 | 0.955-1.147 | 0.332 |
| Abbreviations: HR= hazard ratio; CI=confidence interval; SOFA=sequential organ failure assessment; BSI= bloodstream infection; IAI=intra-abdominal infection; UTI= urinary tract infection; CVC=central venous catheter; NF-GNR= Non fermentative Gram negative rods. | | | |
