# Supplementary figures and images for: Epidemiological characteristics and management of Gram-negative bacteraemia in different immunocompromised hosts: Observational single-center study
Source: PLoS One. 2025 Jul 7;20(7):e0327535. doi: 10.1371/journal.pone.0327535 (PMC12233224; doi:10.1371/journal.pone.0327535)

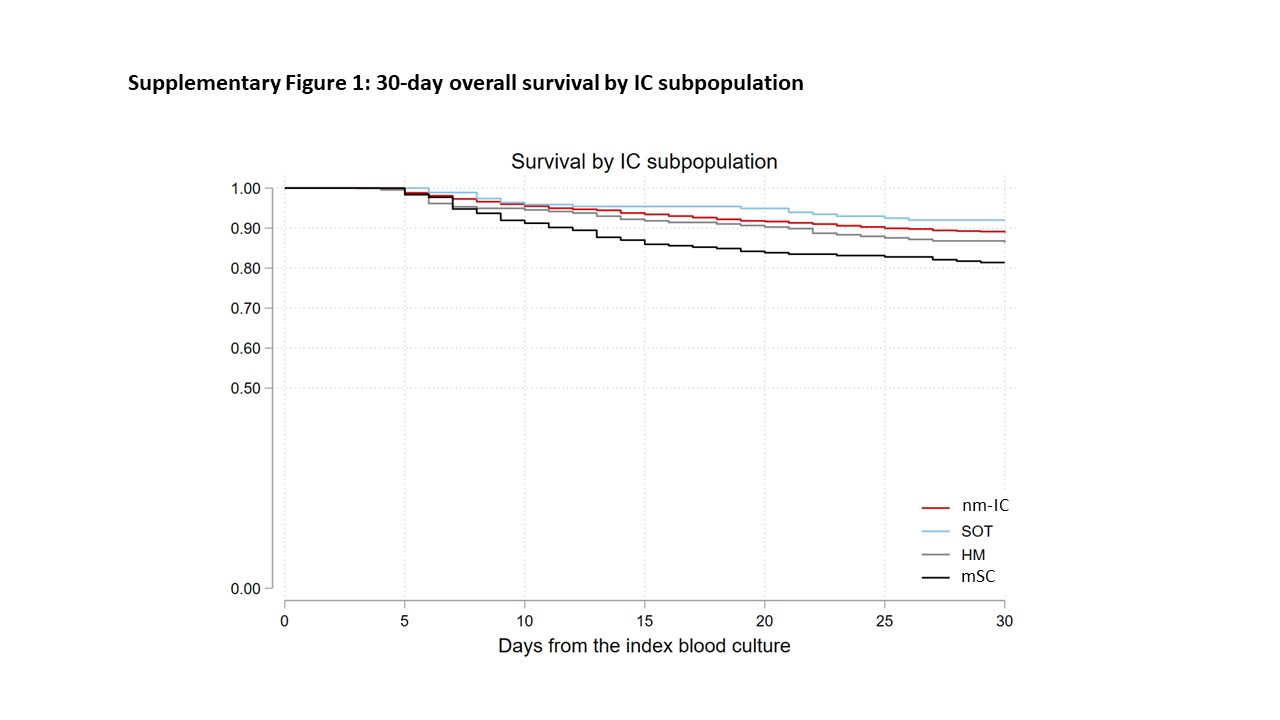

Supplement: S1 Fig — IC = immunocompromised condition; nm-IC = non major immunocompromised condition; SOT = solid organ transplantation; HM = haematological malignancies; mSC = metastatic solid cancer. (JPG) [file pone.0327535.s013.jpg]

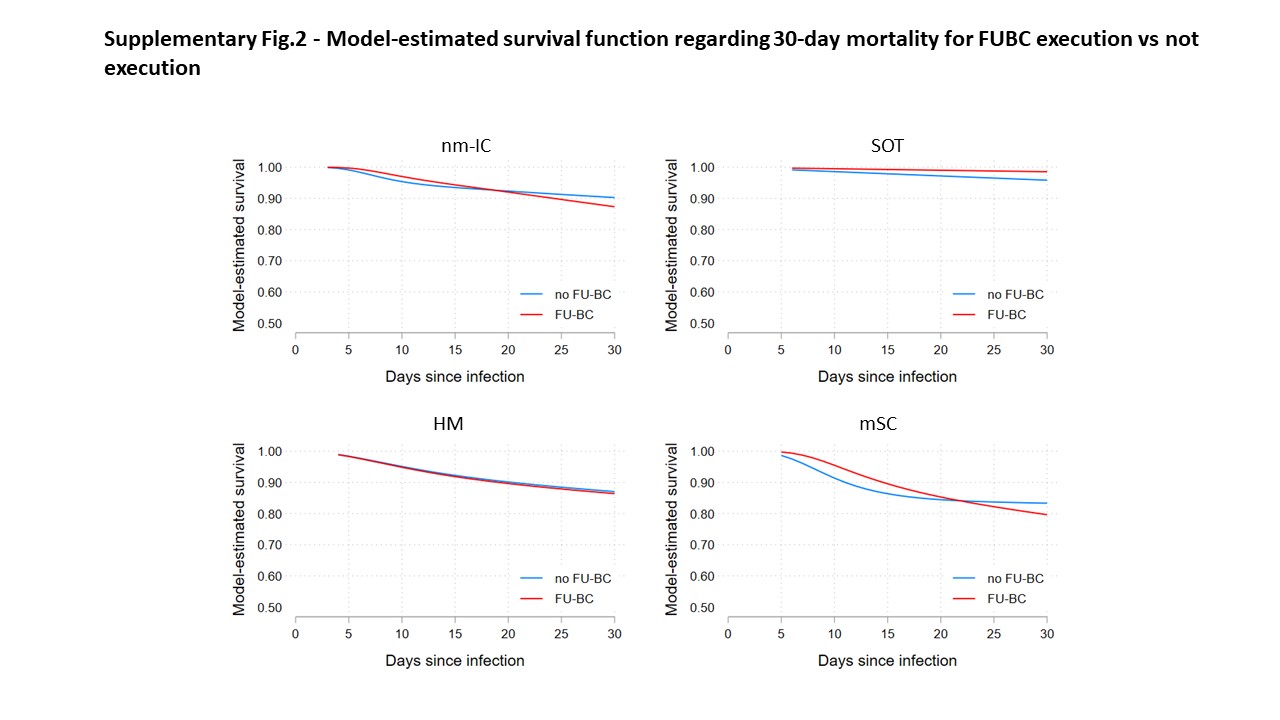

Supplement: S2 Fig — FUBC = follow up bloodcultures; nm-IC = non major immunocompromised condition; SOT = solid organ transplantation; HM = haematological malignancies; mSC = metastatic solid cancer. (JPG) [file pone.0327535.s014.jpg]
